# Modeling of Stripe Patterns in Photosensitive Azopolymers

**DOI:** 10.3390/polym12040735

**Published:** 2020-03-26

**Authors:** Bharti Yadav, Jan Domurath, Marina Saphiannikova

**Affiliations:** Leibniz-Institut für Polymerforschung, Hohe Straße 6, 01069 Dresden, Germany; yadav@ipfdd.de (B.Y.); domurath@ipfdd.de (J.D.)

**Keywords:** azopolymers, surface patterning, viscoplastic modeling

## Abstract

Placed at interfaces, azobenzene-containing materials show extraordinary phenomena when subjected to external light sources. Here we model the surface changes induced by one-dimensional Gaussian light fields in thin azopolymer films. Such fields can be produced in a quickly moving film irradiated with a strongly focused laser beam or illuminating the sample through a cylindrical lens. To explain the appearance of stripe patterns, we first calculate the unbalanced mechanical stresses induced by one-dimensional Gaussian fields in the interior of the film. In accordance with our orientation approach, the light-induced stress originates from the reorientation of azobenzenes that causes orientation of rigid backbone segments along the light polarization. The resulting volume forces have different signs and amplitude for light polarization directed perpendicular and parallel to the moving direction. Accordingly, the grooves are produced by the stretching forces and elongated protrusions by the compressive forces. Implementation into a viscoplastic model in a finite element software predicts a considerably weaker effect for the light polarized along the moving direction, in accordance with the experimental observations. The maximum value in the distribution of light-induced stresses becomes in this case very close to the yield stress which results in smaller surface deformations of the glassy azopolymer.

## 1. Introduction

Azobenzene-containing materials are known as highly versatile systems which have many applications in different fields. These materials can be used as molecular switches [1,2,3] and fancy molecular motors [4], because the parent azobenzene exists in two isomeric forms, which can be interchanged using light of appropriate wavelength or heat [5]. The light responsive materials made of azopolymers are used in liquid crystalline technology [6,7,8,9] and as smart polymers for soft-robotics [10,11,12]. Some natural phenomena like fly trapping plants [13] and caterpillar-like crawling [14] can be mimicked by designing micro-robotic systems incorporating azobenzene chromophores.

Placed at interfaces, azobenzene-containing materials show extraordinary phenomena when subjected to external light fields. For example azopolymers can be easily deformed in the presence of polarized light and the deformations can be directed by the light polarization, for reviews see [15,16]. The light-induced deformations occur even below the glass transition temperature, because the light-induced stress can be much larger than the yield stress as predicted theoretically [17,18,19] and proved experimentally [20,21,22]. As was discovered 25 years ago by two independent groups [23,24], the surface relief gratings can be generated in the thin azopolymer films by applying the light with spatially varying intensity or polarization pattern. The phenomenon is reversible; it is even possible to switch between different surface reliefs interactively [20,25] or erase them fast by shifting polarization patterns with time [26]. This opens a way for new spectacular applications like transport systems for adsorbed liquid droplets and colloids. Moreover, by changing the polarization of a probe beam it is possible to switch the fine structure of the diffraction spot [27,28], which is potentially useful in photonic devices. Further, as light intensity and polarization can be controlled remotely and precisely, the azopolymers can be used as the healing agents for the cracks in the conductive materials in the presence of light stimulus which guides the direction of healing [29].

All these phenomena have their origin in the ability of azobenzene to isomerize between the trans and cis state, followed by orientation of isomers in the presence of polarized light. Due to the angle-selective absorption of photons by the trans-isomers, their long axes preferably orient in the direction perpendicular to the light polarization after a number of photo-isomerization events. As a result, the light-induced orientation has a pronounced angular dependence with only a few trans isomers lying parallel to the light polarization. The circularly polarized (and even unpolarized) light induces orientation of the chromophores along the beam propagation axis. This orientation phenomenon has been called “angular hole burning effect” [30,31]. It can be described by introduction of the effective orientation potential, as has been shown in a series of our papers [19,32]. Based on this orientational picture, the generation of light-induced stress can be predicted in two ways. One approach is to use molecular dynamics simulations where, because of the inter-molecular interaction the reorientation of the azobenzene chromophores is finally transferred to the reorientation of the main chain. The light-induced stress can then be estimated from the orientational order parameters using the Giesekus relation [33]. Secondly, the main chain can be modelled as an ensemble of rod-like Kuhn segments and for such an ensemble the light-induced stress can be calculated analytically from the effective orientation potential [19,32].

The second approach has been successfully applied to explain the directional deformations of the individual azopolymer posts [29,34] and micropillars [35]. In particular, it has been observed in study [29] that an epoxy based azopolymer square post deforms directionally in the presence of polarized light. In agreement with this experiment we predict that the post deforms in the direction of polarization for linearly polarized light and in radial direction for circularly polarized light [32]. Presently we would like to test our approach on directional deformations taking place in the upper layer of thin azopolymer films. Some years ago a puzzling phenomenon was discovered by Ambrosio et al. [36]. When a thin azo-polyurethane film is moved under a highly focused linearly polarized Gaussian beam in two different directions—parallel and perpendicular to light polarization, the film surface deforms differently. In case of parallel movement, the material deforms in the direction of light polarization by making an elongated protrusion [36]. In case of the movement perpendicular to the polarization, a groove was observed. The authors checked that in both cases the overall volume stays conserved. Interestingly, the depth of the grooves has been found to be much larger than the height of the protrusions. No interpretation of this puzzling phenomenon has been found till now.

In this paper we will offer a tentative explanation for the formation of protrusions and grooves for an azopolymer sample moved along and perpendicular to the light polarization. For that we calculate the light-induced stress caused by linearly polarized Gaussian beam in a quickly moving film, using the method described in detail in our previous paper [32]. The glassy azopolymer is considered as viscoplastic material having a constant yield stress. Using the finite element software ANSYS, the stripe-like deformations of thin polymer films can be calculated by applying the light-induced stress.

## 2. Orientation Approach

### 2.1. Effective Potential

The time dependent orientation process of azobenzene chromophores is very well defined by the effective orientation potential which acts on each azobenzene, both in the trans and cis state [19]:

(1) $$u_{\mathrm{eff}}=V_{0}\cos^{2}\left(\theta \right)$$

Here, $V_{0}$ is the strength of the orientation potential which is proportional to the light intensity *I*, θ is the angle between the long axis of trans-isomer and the electric field vector E of the light. The strength of potential $V_{\infty }$ in the steady state differs only slightly from its value $V_{0}$ at the initial stage of illumination, see Supporting Information in [19]. Therefore, in Equation (1) it is safe to consider a constant value $V_{0}$ which is defined by optical and viscous characteristics of the material. The azopolymer samples demonstrate directional deformations in the presence of linearly polarized light, which means the effective orientation potential acting on the azobenzene chromophores is transferred to the polymer backbone. To describe this effect, we model an azopolymer as a main chain consisting of a number of rigid Kuhn segments and *m* azobenzene chromophores rigidly attached to each Kuhn segment. To model different chemical architectures, the shape factor $q=\left[3\langle \cos^{2}\alpha \rangle -1\right]/2$, where α is the angle between the long axes of trans-isomer and Kuhn segment, is introduced for the azopolymers with isotropic azimuthal distribution of chromophores around the main chain [17,37]. The change of photo-mechanical behavior is predicted when crossing a neutral line with q=0: the sample made from the molecules with average $\langle \cos^{2}\alpha \rangle <1/3$ ($\alpha >54.7^{\circ }$) expands along the light polarization vector E and contracts otherwise. Using the shape factor, the effective orientation potential for the azopolymers can be recalculated as [32]:

(2) $$U_{\mathrm{eff}}=qmV_{0}\cos^{2}\left(\theta \right).$$

Note that θ is now the angle between the unit orientation vector u of the Kuhn segment and the unit vector $\hat{E}=E/\left|E\right|$ of light polarization: $\cos\theta =\hat{E}\cdot u$.

### 2.2. Light-Induced Stress

We study here the side chain azo-polyurethane in which the azobenzene chromophores are attached preferentially perpendicularly to the main chain and the shape factor is negative, q<0. Therefore, the effective potential (2) forces the Kuhn segments to orient in the direction parallel to the light polarization. This induces unbalanced stresses in the azopolymer sample [32]:

(3) $$\tau =-3nkT\langle uu\rangle +nkT\delta -\frac{n}{2}\langle u\frac{\partial U_{\mathrm{eff}}}{\partial u}+\frac{\partial U_{\mathrm{eff}}}{\partial u}u\rangle$$

Here, τ is the total stress tensor, *n* is the number density of the Kuhn segments, δ is the unit tensor, *k* is the Boltzmann constant, *T* is the absolute temperature and $\frac{\partial }{\partial u}u=\delta -uu$ [38]. The stress induced by the effective orientation potential is given by the last term in the above equation. For linearly polarized light this stress can be expressed by the following equation [32]:

(4) $$\tau_{\mathrm{light}}=-nqmV_{0}\left[\hat{E}\hat{E}\cdot \langle uu\rangle +\langle uu\rangle \cdot \hat{E}\hat{E}-2\hat{E}\hat{E}:\langle uuuu\rangle \right]$$

Here, 〈uu〉 and 〈uuuu〉 are the second and fourth order orientation tensors which describe the average orientation state of an ensemble of backbone segments. The fourth order tensor can be calculated from the second one using an appropriate closure approximation. Note that $\tau =\tau_{\mathrm{light}}$ for the isotropic state in the beginning of illumination, when 〈uu〉=δ/3. The tensor 〈uuuu〉 can be calculated for the isotropic state exactly using the linear closure [39].

**Figure 1:**
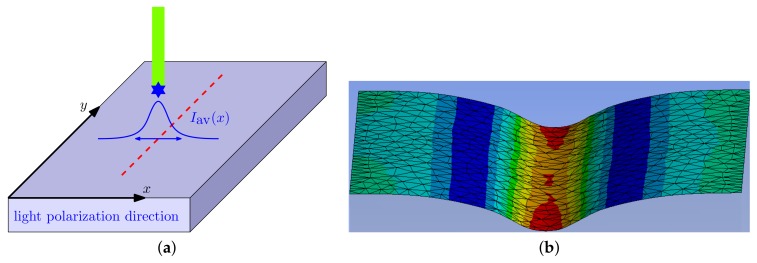
(**a**) The laser is moved (denoted with red dashes) in the *y* direction perpendicular to light polarization aligned along the *x* direction. (**b**) The deformations for the upper surface of the material in the presence of *x* polarized light. $\tau_{\max}=50$ MPa. The beam radius $w=2\sqrt{10}$ μm.

**Figure 2:**
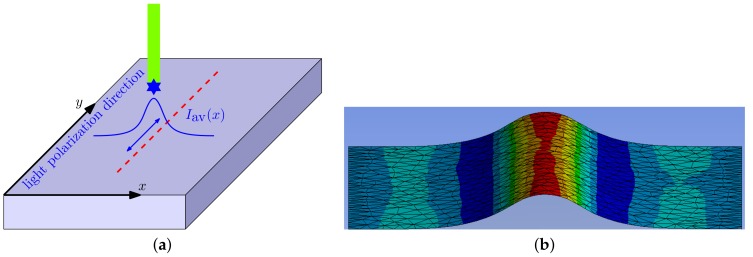
(**a**) The laser is moved (denoted with red dashes) in the *y* direction parallel to light polarization aligned along the *y* direction. (**b**) The deformations for the upper surface of the material in the presence of *y* polarized light. $\tau_{\max}=50$ MPa. The beam radius $w=2\sqrt{10}$ μm.

In the experiment of Ambrosio et al. [36] the direction of light polarization was kept constant but the film was moved in different directions. This is equivalent to moving the beam with different polarizations in the same direction, here along the *y* axis, as shown in the Figure 1a and Figure 2a. With such an assignment the stress tensor can be written in a simplified diagonal form which appears due to the axial symmetry of the system with respect to the polarization vector E [19,32].

For light polarized along *x* axis

(5) $$\hat{E}\hat{E}=\left[\begin{array}{ccc} 1 & 0 & 0\\ 0 & 0 & 0\\ 0 & 0 & 0 \end{array}\right]\;\mathrm{and}\;\tau =\tau \left[\begin{array}{ccc} 1 & 0 & 0\\ 0 & -\frac{1}{2} & 0\\ 0 & 0 & -\frac{1}{2} \end{array}\right]$$

For light polarized along *y* axis

(6) $$\hat{E}\hat{E}=\left[\begin{array}{ccc} 0 & 0 & 0\\ 0 & 1 & 0\\ 0 & 0 & 0 \end{array}\right]\;\mathrm{and}\;\tau =\tau \left[\begin{array}{ccc} -\frac{1}{2} & 0 & 0\\ 0 & 1 & 0\\ 0 & 0 & -\frac{1}{2} \end{array}\right]$$

When the glassy azopolymer sample is put under light illumination for a long time (in the order of minutes), the stress tensor decays gradually with time due to a slow reorientation of backbone segments [32]. However, for the case when the thin azopolymer films are moved very fast under the Gaussian beam, the exposure time for one spot on the film is very short (in the order of seconds). In particular, the inscription time for a 26 μm long stripe was about 2 min and 22 s [40]. Therefore, the deformation of a fast moving film is mostly caused by the light-induced stress generated at the very beginning of illumination.

The magnitude of the stress tensor τ can be extracted from Equation (4) assuming intitally isotropic orientational state with $\langle u_{x}u_{x}\rangle =1/3$ and $\langle u_{x}u_{x}u_{x}u_{x}\rangle =1/5$:

(7) $$\tau =-\frac{4nqmV_{0}}{15}>0$$

To estimate $V_{0}$, which depends on the light intensity, we consider here a Gaussian beam with the following intensity profile:

(8) $$I\left(x,y\right)=I_{0}\exp\left(-2\frac{{\left(x-a\right)}^{2}+y^{2}}{w^{2}}\right)$$

where $I_{0}$ is the intensity at the beam center (x=a, y=0), *w* is the beam radius (=size of the laser spot) at which intensity falls to $1/e^{2}$ of its maximal value $I_{0}$. For a fast movement in the *y* direction, the intensity can be averaged over all possible *y* inside the laser spot which results in the following expression:

(9) $$I_{\mathrm{av}}\left(x\right)=CI_{0}\exp\left(-\frac{2{\left(x-a\right)}^{2}}{w^{2}}\right)$$

where $C=\sqrt{\pi /2}\mathrm{erf}\left(\sqrt{2}\right)/2\approx 0.60$. Hence $\tau ∼V_{0}∼I_{\mathrm{av}}$ will have the same *x* dependence as the average intensity of the beam:

(10) $$\tau \left(x\right)=\tau_{0}\exp\left(-\frac{2{\left(x-a\right)}^{2}}{w^{2}}\right)$$

where $\tau_{0}$ is the magnitude of the stress tensor at the stripe center (x=a).

### 2.3. Volume Force and Traction

Using the orientation approach, we are able to predict the light-induced stress field in the azopolymer sample. However, in mechanical applications it is not possible to apply the forces to a solid body in the form of the stress tensor. They can be either applied to the interior of the body in the form of so called volume forces or to the surface of the body as surface traction [41]. Let us first consider the volume force formalism, as it easily explains why *x* and *y* polarized light beams induce different deformations. The external force per unit volume can be calculated from the stress tensor as

(11) f=−∇·τ

with the *k* component given by

(12) $$f_{k}=-\sum_{i}\frac{d\tau_{ik}}{dx_{i}}$$

The stress components in both cases have only *x* dependence, see Equations (5), (6) and (10), thus only the *x* component of the volume force has a non-zero value.

For light polarized in *x* direction

(13) $$f_{x}=-\frac{d\tau }{dx}=4\tau_{0}\frac{x-a}{w^{2}}\exp\left(-\frac{2{\left(x-a\right)}^{2}}{w^{2}}\right)$$

For light polarized in *y* direction

(14) $$f_{x}=\frac{1}{2}\frac{d\tau }{dx}=-2\tau_{0}\frac{x-a}{w^{2}}\exp\left(-\frac{2{\left(x-a\right)}^{2}}{w^{2}}\right)$$

**Figure 3:**
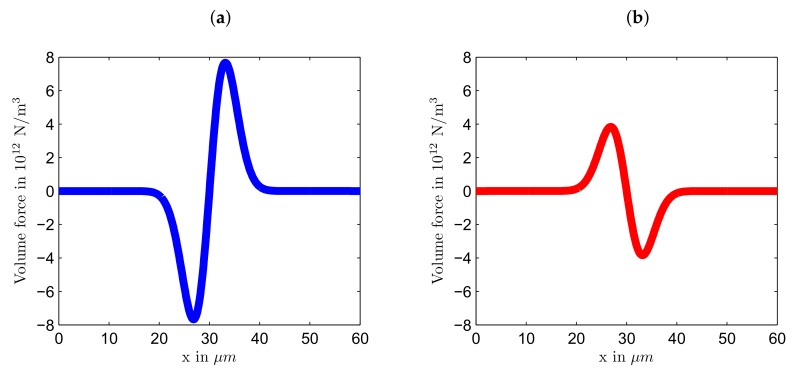
The *x* component of the volume force: (**a**) stretching for light polarized in *x* direction and (**b**) compressive for light polarized in *y* direction. $\tau_{0}=40$ MPa. The beam radius $w=2\sqrt{10}$ μm.

The magnitude of volume force for light polarized in *x* direction is twice the magnitude of the volume force for light polarized in *y* direction. Hence, we can expect that the magnitude of deformations caused by the light polarized in *x* direction will be larger than the deformations caused by the light polarized in *y* direction. The opposite sign of the volume force in Equations (13) and (14) predicts that the deformations will be in opposite direction for two different polarizations. As can be seen from Figure 3a, *x* polarized light produces the stretching force, as it is positive at x>a=30 μm and negative at x<a. Contrary, *y* polarized light results in the compressive force along the *x* direction, see Figure 3b.

Interestingly, the formalism of volume force predicts that no effect should be observed for the light polarized at a particular angle to the moving direction *y*. This angle can be found by considering the light-induced stress tensor in the principle axes and then rotating it into the laboratory coordinate system. The volume force for an arbitrary angle φ between the light polarization and the *x* axis is

(15) $$f_{x}=-\frac{1}{2}\frac{d\tau }{dx}\left(3\cos^{2}\varphi -1\right)$$

Hence, the force becomes equal to zero at $\varphi =54.7^{\circ }$, i.e., at $35.3^{\circ }$ to the moving direction.

## 3. Material Modeling

The deformations in glassy azopolymer materials are well described by a viscoplastic material model, in particular by the Bingham solid model [42]:

(16) $$\tau =\left\{\begin{array}{cc} 2G\epsilon_{\mathrm{el}}, & \tau_{\mathrm{eq}}\leq \tau_{\mathrm{yield}}\\ \tau_{\mathrm{yield}}+2\eta {\dot{\epsilon }}_{\mathrm{pl}}, & \tau_{\mathrm{eq}}>\tau_{\mathrm{yield}} \end{array}\right.$$

Here, *G* is the shear modulus of the azopolymer, η is the viscosity of plastic flow, $\tau_{\mathrm{yield}}$ is the yield stress, $\epsilon_{\mathrm{el}}$ is the elastic strain tensor and ${\dot{\epsilon }}_{\mathrm{pl}}$ is the rate of plastic strain tensor. At the yield point $\tau_{\mathrm{eq}}=\tau_{\mathrm{yield}}$, the yield stress is $\tau_{\mathrm{yield}}=2G\epsilon_{\mathrm{el}}$ and $\tau_{\mathrm{eq}}=\sqrt{\frac{3}{2}\tau :\tau }$ is the von Mises equivalent stress. Existence of the yield threshold in glassy azopolymers has been confirmed by the experimental study [43], where no light-induced surface deformation was found for the case when only every 200th monomer was occupied by an azobenzene. Below the yield stress the glassy polymers deform elastically, above the yield stress they start to develop the plastic deformations which considerably overcome the elastic deformation after a long irradiation time. For plastic deformations the volume is conserved. The material parameters are chosen as in our last paper [32]: the Young’s modulus E=3G=1 GPa and $\tau_{\mathrm{yield}}=10$ MPa.

To model stripe-like deformations on the surface of azopolymer sample, we used the Perzyna model (a counterpart of the Bingham solid model) in the finite element software ANSYS with strain rate hardening parameter m=1 and the material viscosity parameter γ=0.1 s${}^{-1}$. The viscosity of plastic flow can be estimated as $\eta =\tau_{\mathrm{yield}}/3\gamma$ [32]. Together with the light-induced stress, it defines the rate of plastic deformation which appears to be rather high for strongly focused Gaussian beams.

## 4. Modeling Results

First, we checked the sign and the strength of the light-induced effect. For that a finely meshed cubic sample with the edge length of 30 μm was generated. The following boundary conditions were applied: (1) the bottom face of the sample was restricted from moving in all three directions similar to the experiments where the azopolymer sample was “glued” to the substrate surface due to a strong adhesion [29,44], (2) the upper surface was free to move in all three directions and (3) all other sides were restricted from moving in the normal direction. While it proved difficult to apply the volume forces in the ANSYS software, which operates only with the forces applied to the nodes (and thus the total force changes with the remeshing), we chose an alternative way of applying the light induced stress. For that we proved, using a home-made finite element software, that the stretching volume force (13) applied to the elastic solid at previously mentioned boundary conditions induces the same deformation field as the traction force acting normally on the upper sample surface in negative direction (inwardly): $t_{z}\left(x\right)=-\tau_{\max}\exp\left(-\frac{2{\left(x-a\right)}^{2}}{w^{2}}\right)$ with $\tau_{\max}=\tau_{0}$. Similarly, the contractive volume force (14) is equivalent to the traction force acting in positive direction (outwardly): $t_{z}\left(x\right)=\tau_{\max}\exp\left(-\frac{2{\left(x-a\right)}^{2}}{w^{2}}\right)$ with $\tau_{\max}=\tau_{0}/2$. In both cases, to test the strength of effect, we applied different maximal tractions $\tau_{\max}$ at the stripe center, ranging from 40 MPa to 80 MPa. Figure 1b presents exemplarily how the azopolymer surface will deform in the presence of light polarized in the *x* direction at $\tau_{\max}=50$ MPa and $w=2\sqrt{10}$ μm after 5 s. In accordance with the experiment of Ambrosio et al. [36], the deformed surface looks like a stripe-like well with uplifted ends. Figure 2b shows an example of the deformed surface in the presence of light polarized in the *y* direction at the same conditions. Again, in accordance with the experiment, a stripe-like protrusion can be observed.

Comparing modeling results with the depth (∼0.2 μm) and height of stripes (∼0.05 μm) inscribed experimentally [36], we realized that traction forces with $\tau_{\max}$ above 50 MPa considerably overpredicted the strength of the effect. The surface deformations were far above 10 μm and did not disappear at the sample boundaries in *x* direction. Therefore, in an attempt to reproduce the experiment of Ambrosio et al. [36] not only qualitatively but also quantatively, we chose another sample (0≤x≤60 μm, 0≤y≤15 μm, 0≤z≤15 μm) which was twice as long in *x* direction and twice as short in two other directions. The latter adjustment was made to keep the computational effort in reasonable limits. The boundary conditions were the same as for the first sample.

**Figure 4:**
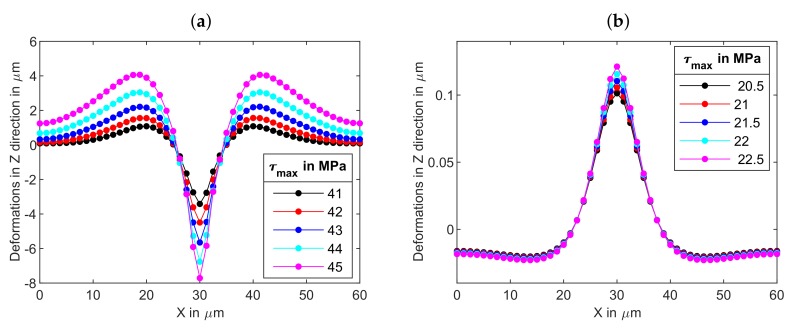
The deformations in *z* direction for light polarized (**a**) in *x* direction and (**b**) in *y* direction at different values of maximal traction shown in the legend. The film dimensions are 0≤x≤60 μm, 0≤y≤15 μm, 0≤z≤15 μm and the beam radius $w=2\sqrt{10}$ μm.

Figure 4a presents the surface profiles along the *x* axis for light polarized in *x* direction for different values of $\tau_{\max}$ after 20 s of stress application. In the absence of light z=0 corresponds to the free surface of azopolymer sample and z<0 to its interior. It can be seen that the deformations in the *z* direction were maximum at the center of the sample (at the center of the stripe) and minimum at the *x* boundaries. Similar to experimental observation [36], formation of a well at the sample center was accompanied by two small hills to the both sides of the center. The depth of the well and height of the small hills rapidly grew with the increase of light intensity: the well was about 3 μm deep for $\tau_{\max}=41$ MPa and 8 μm for 45 MPa. To model surface changes caused by light polarized in *y* direction, we applied twice smaller values of $\tau_{\max}$. This should have corresponded to the same range of light intensities, as explained above. As can be seen from Figure 4b, light polarized in *y* direction caused a much smaller effect. A low hill was formed at the center of the stripe and was accompanied by two shallow valleys at the sides. The height of the hill was about 0.1 μm at $\tau_{\max}=20.5$ MPa, it increased only slightly with further growth of $\tau_{\max}$. Such difference in behavior can be explained by the fact that light polarized in *y* direction induced the stress field which was considerably closer to the yield stress of 10 MPa than the field induced by *x* polarized light. We remind here that the plastic deformations were caused by the excess equivalent stress and quickly died when the light-induced stress approached the yield value.

**Figure 5:**
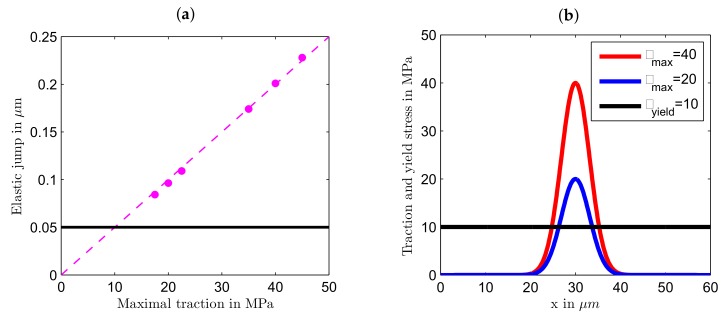
(**a**) The magnitude of the elastic jump $\Delta z_{\mathrm{el}}$ at the stripe center is proportional to $\tau_{\max}$, both for *x* and *y* polarized light. The black line shows $\Delta z_{\mathrm{el}}$ at the yield stress. (**b**) The film area in which the stresses exceed the yield stress rapidly shrinks with the decrease of $\tau_{\max}$. The beam radius $w=2\sqrt{10}$ μm.

**Figure 6:**
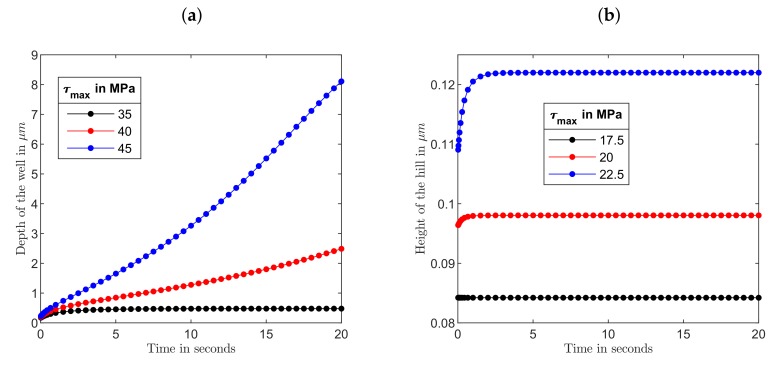
Time-dependent deformations in *z* direction for light polarized (**a**) in *x* direction and (**b**) in *y* direction at different values of maximal traction shown in the legend. The beam radius $w=2\sqrt{10}$ μm.

Comparing the depth of the well with the height of the hill, one can see that they differed nearly 30 times at the smallest $\tau_{\max}$. However, such a high difference in the strength of effect was found after 20 s of stress application. It could be that at shorter “illumination” times the difference would diminish and therefore we investigated time-dependent deformations in *z* direction at the stripe center. Here we found a very interesting effect. When the light wasswitched on, one observed first an instanteneous elastic jump, the magnitude of which in the Perzyna model is proportional to the value of maximal traction: $\Delta z_{\mathrm{el}}∼\tau_{\max}$, both for light polarized in *x* and *y* directions, see Figure 5a. The elastic jump $\Delta z_{\mathrm{el}}=0.05$ μm proportional to the value of yield stress, see the black line in Figure 5a, seemed to be more plausible physically; otherwise, the jump became rather large at strong irradiation. However, this is very difficult or perhaps even impossible to prove experimentally. Interestingly, after an inital elastic jump the plastic deformations developed differently, see Figure 6: they either died after a couple of seconds at $\tau_{\max}\leq 35$ MPa or started to grow persistently at $\tau_{\max}\geq 40$ MPa.

Presumably, this was caused by a fast equilibration of initially unbalanced stresses, which is only possible at small enough values of $\tau_{\max}$, when a large part of the film was located outside the area in which the stresses exceeded the yield stress, see Figure 5b. A similar difference in behaviour was observed for surface relief gratings inscribed on the azopolymer film by two types of interference patterns [25]: the grating growth saturated after 10–15 min for intensity patterns but no saturation was found for the gratings produced by polarization patterns. Hence, we expect that for the polarization patterns, characterized by a constant intensity along the film surface, the stress field was everywhere above the yield stress, while for the patterns with sinusoidally varying intensity the stress field should be below the yield stress in some areas.

Let us return to the stripe patterns generated by a Gaussian beam in a fast moving film [36]. Here the inscription rate was about 1 μm per 5 s [40]. If we compare the modeling results after 5 s of the stress application, see Figure 6, the ratio of the well depth to the hill height was considerably smaller changing from 5.4 to 13.5 when $\tau_{\max}$ increased from 35 MPa to 45 MPa for *x* polarized light and half of these values for *y* polarized light. This comparison was done for the elastic jump proportional to the value of maximal traction as predicted in the Perzyna model, see Figure 5a. Making a correction on the elastic jump proportional to the yield stress, approximately twice higher ratios of the well depth to the hill height would be obtained. In any case, our viscoplastic modeling showed that it was possible to find a combination of parameters (beam radius, maximal traction, illumination time) at a chosen thickness of azopolymer film (15 μm) which provided a strength of the effect, comparable with that found in the experiment.

**Figure 7:**
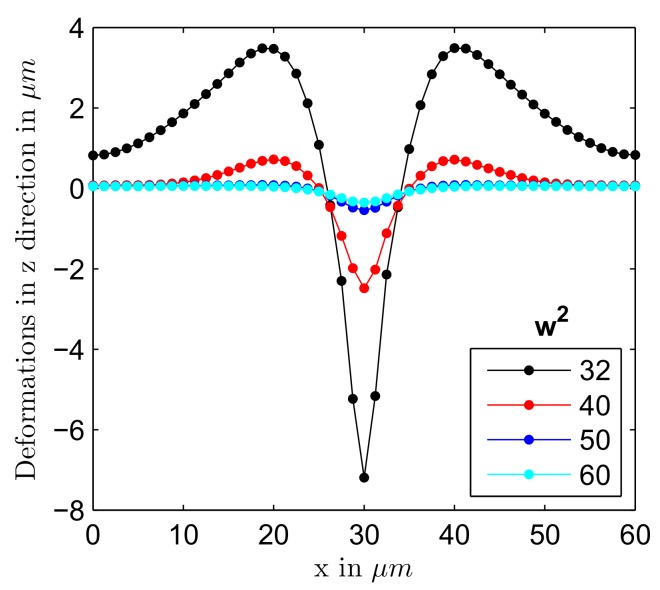
The deformations in *z* direction for light polarized in *x* direction at different values of beam radius shown in the legend.

Not all of these parameters were independent from each other. Usually, the source of laser light had a constant power. For example, the power of the laser was set to 12 μW in the experiment of Ambrosio et al. [36]. Focusing a beam to the spots with decreasing radius can lead to increase of the intensity at the beam center by orders of magnitude. Figure 7 illustrates a possibility to manipulate the strength of the effect by changing the radius *w* of the *x* polarized beam. The maximal traction was equal to 40 MPa at $w=2\sqrt{10}$ μm and it was adjusted for other beam radii in such a way that the total power transmitted by the Gaussian beam stayed constant: $P={\;\int \int \;}_{-\infty }^{\infty }I\left(x,y\right)dxdy=1/2\pi I_{0}w^{2}$, where the intensity profile is given by Equation (8). One can see that the well depth and the hight of accompanying hills were very sensitive to small adjustments in the size of the laser spot. In particular, weaker focusing results in a quick disappearance of the surface modulation, which can be explained by the drop of light-induced stress below the yield stress in the most part of the illuminated area.

Modeling results presented in this section demonstrate that the orientation approach is capable of explaining not only homogeneous deformations of the individual azopolymer posts [29,34] and micropillars [35] but also inscription of stripe-like patterns onto the surface of azopolymer films [36]. After discovery of surface relief gratings in 1995 [23,24], a great variety of surface patterns has been inscribed on films produced from different azobenzene-containing polymers. In the next section we discuss two experiments that are most relevant to the present modeling attempt.

## 5. Discussion

The experiment [36], which we discussed in detail here, was performed with the azo-polyurethane film having very high glass transition temperature $T_{g}$ of 183 ${}^{\circ }$C. A similar experiment was carried out with a convential azopolymer, characterized by a considerably lower $T_{g}∼$ 100 ${}^{\circ }$C. Five adjacent grooves were inscribed one after another on the azopolymer sample by moving it perpendicular to the light polarization of the Gaussian beam and then shifting the sample by 1 μm sideways [45]. When the sample is moved parallel to the light polarization, a small grating of adjacent protrusions could be inscribed. The height of protrusions was approximately four times smaller than the depth of the grooves, which is comparable with the strength of effect in the azo-polyurethane film [36]. This is a bit unexpected as both polymers were irradiated with a strongly focused Gaussian beam that can easily heat the surface of azopolymer sample. For example, the surface of poly(disperse orange 3) can be heated above its $T_{g}≃$ 120 ${}^{\circ }$C after 60 s of the white light irradiation at 1.8 W/cm${}^{2}$, see Figure S5 in Ref. [46].

It is important to point out that the glass transition temperature of some azopolymers can be decreased below the room temperature by irradiation with ultraviolet light [47,48,49]. However, such polymers have special molecular features that facilitate a photoinduced solid-to-liquid transition. In particular, they contain azobenzene-type chromophores and long alkyl chains as the spacer and the tail of a side-chain. Molecular structures of the side-chain azopolymers used by Ambrosio et al. [36], Ambrosio et al. [45] are very close to those for which no decrease of Tg was reported under irradiation with ultraviolet light in [48]. Therefore, we do not expect a decrease of $T_{g}$ for either of the azopolymers under irradiation, especially as the visible light was used to inscribe the stripes.

As we discussed in Ref. [18], the orientation effects should disappear above the glass transition and therefore we do not expect that differently polarized beams will inscribe different structures above this transition. Indeed, as was reported in Ref. [46], when a high intensity laser is moved over the sample surface, the wrinkles in a fine structure are always parallel to the direction of laser movement and are independent on the light polarization. The wrinkling direction is fully guided by alignment of soft/hard boundary between illuminated and non-illuminated areas. Estimation of the light intensity in the experiments [36,45] provides extremely high values which exceed 100 W/cm${}^{2}$ at the beam center. Nevertheless, appearance of grooves for *x* polarized light and protrusions for *y* polarized light can only indicate that both azopolymers, although heated, stay below their $T_{g}$ due to fast movement of the sample under Gaussian beam.

Another relevant experiment was performed in 1999 by the Tripathy group [50], who put a poly(disperse orange 3) film under a one-dimensional Gaussian beam with two different polarizations: along the direction of intensity gradient and perpendicular to it. Such beam can be obtained by using a cylindrical lens instead of a usual spherical one. The intensity profile of cylindrical Gaussian beam can be described by Equation (9). The intensity at the beam waist center was about 0.3 W/cm${}^{2}$ and thus the average intensity was considerably lower than in the experiments of Ambrosio et al. [36], Ambrosio et al. [45]. Therefore, to induce noticable changes, the surface of azopolymer sample was irradiated for a rather long time of 70 min. For the light polarization along the direction of intensity gradient (corresponds to *x* polarized light in our notation), the film was deforming in the direction of light polarization. In this case an elongated groove is formed on the azopolymer surface, the shape of which is similar to that predicted by us for *x*-polarized light. In spite of a long irradiation time, the maximum depth modulation was about 20 nm, which is rather low. For the light polarization orthogonal to the intensity gradient (corresponds to *y* polarized light in our notation), no appreciable surface modulation but only photoinduced birefringence was detected. Considering our modeling results, both findings are quite expectable. The stress field induced by *x* polarized light is presumably only slightly above the yield stress, which explains so low surface modulation. For *y* polarized light the light-induced stresses should fall below the yield stress. In the latter case the reorientation of azobenzenes and backbones is not able to induce noticable changes on the surface of azopolymer sample.

## 6. Outlook

In the present study we gained a deeper understanding of the mechanical action of one-dimensional Gaussian fields which are characterized by a constant direction of light polarization. In accordance with earlier reports [16,50], our orientation approach predicts that such fields should induce the highest surface modulations when the light polarization is directed along the intensity gradient of the cylindrical Gaussian beam. At high enough intensities, the light polarization orthogonal to the intensity gradient can also induce surface changes, though they are predicted to be considerably weaker. Additionally, we expect that no surface modulation should arise at a particular angle between the moving direction of the beam and the light polarization.

A straightforward verification of the orientation approach will be its application to the light fields generated in a strongly absorbing medium. This effect was neglected in the present modeling due to extremely high intensities used in insciption of stripe-like patterns, assuming that each sublayer in the film interior receives more than enough photons to excite the available population of azobenzenes. We already applied the orientation approach to a two-dimensional Gaussian beam, strongly absorbed in the elastic azopolymer matrix, using a home-made finite element software. Surface modulations are found to be in accordance with the experimental observations [50]. This promising result inspires us to switch to the modeling of surface relief gratings, at least in the approximation of elastic matrix. Such modeling will help to check the sign and strength of effects caused by three-dimentional light fields. Clearly, it would be desirable to implement a full visco-plastic model for the same fields, however, presently we are hindered by restrictions in mechanical implementations of commercial finite element software.
